# Annual of the Universal Medical Sciences

**Published:** 1891-12

**Authors:** 


*Annual of the Universal Medical Sciences.* Edited by CHARLES E. SAJOUS, M.D. Five Volumes. Philadelphia: F. A. DAVIS. 1891.

This work again deserves the highest praise. A review of it in detail is impossible, but we may say that here the best work up to the date of publication is admirably summarised, and placed before the reader in a most attractive and systematic manner. Illustrations are freely given when necessary to elucidate the text. We notice, with much gratitude, that Dr. Sajous now prints in each volume the list of journals from which the abstracts are made.

